# Homo- and Copolymerizations of Ethylene and Norbornene Using Bis(β-ketoamino) Titanium Catalysts Containing Pyrazolone Rings

**DOI:** 10.3390/polym9070262

**Published:** 2017-06-30

**Authors:** Lixia Pei, San He, Jie Gao, Heng Liao, Haiyang Gao

**Affiliations:** 1 State Key Laboratory of Oil and Gas Reservoir Geology and Exploitation, Southwest Petroleum University, Chengdu 610550, China; peilixia@scut.edu.cn (L.P.); hesan@126.com (S.H.); 2 School of Chemistry and Chemical Engineering, South China University of Technology, Guangzhou 510641, China; 3 School of Materials Science and Engineering, PCFM Lab, GD HPPC Lab, Sun Yat-sen University, Guangzhou 510275, China; gaojie29@mail.sysu.edu.cn (J.G.); liaoheng5@mail.sysu.edu.cn (H.L.)

**Keywords:** bis(β-ketoamino) titanium catalyst, ethylene, norbornene, polymerization

## Abstract

A series of bis(β-ketoamino) titanium complexes containing pyrazolone rings (**1**–**3**) have been synthesized, characterized, and used as precursors for homo- and copolymerization of ethylene and norbornene. The titanium complexes activated with methylaluminoxane (MAO) exhibited good activities for homopolymerization of ethylene (E) to produce linear polyethylenes (PEs). Ethylene–norbornene copolymers (E–N) were also prepared by these catalysts with moderate activities, and influences of ligand substituents and norbornene addition on copolymer microstructure were studied in detail. Microstructure analysis of the E–N copolymers by ^13^C NMR and differential scanning calorimetry (DSC) techniques showed that alternating (ENEN) and isolated (ENEE) norbornene predominately appeared in the copolymer chain, and the NN dyad and NNN triad sequences were also present in the copolymers obtained by the less bulky catalyst **1**.

## 1. Introduction

Cyclic olefin copolymers (COCs) of ethylene (E) with cyclic olefins, especially norbornene (N), representing a new class of amorphous materials has attracted great interest over the past decade because of their remarkable properties, such as high vapor and thermal resistance, excellent optical transparency, and high refractive indexes [1,2,3,4,5]. In fact, these material properties can be precisely controlled by varying monomer composition, sequence distribution, and the chain stereoregularity, which closely depends on the employed catalyst structure. Both the ligand environment and the center metal atom type play a major role in the copolymerization of ethylene and norbornene.

Driven by industrial applications, copolymerizations of norbornene and ethylene have been performed using various transition metal catalysts including titanium [6,7,8,9,10,11,12,13,14,15,16,17,18,19,20,21,22,23,24,25,26,27], zirconium [28,29], nickel [30,31,32,33,34,35,36,37,38], palladium [39,40,41,42,43,44,45], chromium [46,47,48], vanadium [49], and rare earth metal catalysts [50,51]. Group IV metal catalysts, especially titanium based catalysts, receive more attention among these different metal based catalysts. For instance, ethylene–norbornene (E–N) copolymers were first obtained by Kaminsky with metallocene/methylaluminoxane (MAO) [6,7,8], and then half sandwich and constrained-geometry titanium catalysts (CGCs) developed by Dow Chemical were widely used in the copolymerization of ethylene and norbornene [9,10,11,12,13,14]. Usually, random E–N copolymers with high norbornene incorporations up to 70 mol % are obtained with these titanium catalysts. Non-metallocene titanium complexes activated by MAO or modified methylaluminoxane (MMAO) are also used to effectively catalyze copolymerization of ethylene and norbornene [15,16,17,18,19,20,21,22,23,24,25]. In particular, titanium catalysts bearing bis(pyrrolide-imino) [16], bis(imino-indolido) [18], and bis(β-enaminoketonato) ligands [19] are reported to catalyze ethylene and norbornene copolymerization in a living fashion. Although non-metallocene titanium catalysts usually produce alternating E–N copolymers, the bis(α-alkyloxoimine) titanium catalyst facilitates random copolymers with high norbornene incorporation up to 76 mol %, because the electron-negative alkoxide group makes the titanium center prone to be attacks by the norbornene monomer [27]. These differences of the E–N copolymer microstructure highlight the powerful ligand influence on the reactivity of titanium catalysts and the potential reward for continued efforts to uncover new type of ligands and catalysts.

Schiff base derivatives containing pyrazolone are important members of the β-ketoamines ligand family because of their ease of preparation and modification of both steric and/or electronic effects. β-Ketoamine ligands containing the pyrazolone ring are easily coordinated to various metal atoms, and nickel-, cobalt-, and copper-based complexes have been synthesized and used to catalyze olefin polymerizations in the presence of MAO [52,53,54,55,56,57,58,59]. To the best of our knowledge, early transition metal complexes chelating β-ketoamine ligands containing pyrazolone rings have not been synthesized and studied for their catalytic properties. Herein, we have reported novel bis(β-ketoamino) titanium precursors containing pyrazolone rings for homo- and copolymerizations of ethylene and norbornene. The influences of catalyst structure and norbornene addition on copolymerization activity and incorporation of norbornene in copolymers have been investigated in detail. 

## 2. Experiment

All manipulations involving air- and moisture sensitive compounds were performed under dried and purified nitrogen (99.999%) using standard vacuum-line, Schlenk, or dry glovebox techniques.

### 2.1. Materials 

All solvents were purified using standard procedures. *n*-Butyllithium (*n*-BuLi) solution in hexane (2.2 M) was purchased from Aldrich (Saint Louis, MO, USA). Norbornene (bicyclo[2.2.1] hept-2-ene; Acros) was purchased from Acros (Koblenz, Germany). and purified by distillation over potassium metal and used as a solution in toluene. Methylaluminoxane (MAO) solution (10 wt % in toluene) was purchased from Acros. Titanium tetrachloride (TiCl_4_) was distilled prior to use. Ethylene gas (polymerization grade) was further purified by passage through columns of molecular sieves. Other commercial reagents were purchased and directly used without purification. Ligands **HL1** (5-methyl-2-phenyl-4-[(2-phenylamino)-phenylmethylene]pyrazol-3(2H) -one), **HL2** (5-methyl-2-phenyl-4-[(2-o-tolyl)-phenylmethylene]pyrazol-3(2H)-one), and **HL3** (5-methyl-2-phenyl-4-[(2-α-naphthylamino)-phenylmethylene]pyrazol-3(2H)-one) were prepared according the reported method [57].

### 2.2. Measurements

Elemental analyses were performed on a Vario EL microanalyzer (Elementar, Hanau, Germany). Mass spectra for the titanium complexes were measured on a Thermo LCQ DECA XP liquid chromatography (Thermo, San Diego, CA, USA)—mass spectrometry using electrospray ionization. ^1^H NMR spectra were carried out on Mercury-plus 300 MHz NMR spectrometers (Varian, Salt Lake, UT, USA) at room temperature in CDCl_3_ solution for organic compounds. ^13^C NMR spectra of polymers were carried out on a Bruker 500 MHz (Bruker, Rheinstetten, Germany) at 120 °C *o*-C_6_D_4_Cl_2_ solution using solvent as a reference. The norbornene incorporations in the copolymer were calculated from the ^13^C NMR spectra (Bruker, Rheinstetten, Germany). The molecular weight and molecular weight distribution (*PDI* = *M_w_*/*M_n_*) of the polymers at 150 °C were performed on a high-temperature gel permeation chromatography (GPC), PL-GPC 220 instrument (PL, Shropshire, UK) equipped with a differential refractive index (RI) detector. Differential scanning calorimetry (DSC) analyses were conducted with a Perkin Elmer DCS-7 system (Perkin Elmer, Waltham, MA, USA). The DSC curves were recorded at second heating curves at a heating rate of 10 °C/min and a cooling rate of 10 °C/min.

### 2.3. Crystal Structure Determination 

The crystal of Ti complex **2** was mounted on a glass fiber and transferred to a Bruker CCD platform diffractometer (Karlsruhe, Germany). Data obtained with the ω-2θ scan mode was collected on a Bruker SMART 1000 CCD diffractometer (Karlsruhe, Germany) with graphite-monochromated Cu K_α_ radiation (λ = 1.54178 Å) at 293 K. The structure of Ti complex **2** was solved by direct methods using the program SHELXS97 (Göttingen Univeristy, Germany), while further refinement with full-matrix least squares against *F*^2^ was obtained with the SHELXL97 program package. All non-hydrogen atoms were refined anisotropically, and all hydrogen atoms were introduced in calculated positions with the displacement factors of the host carbon atoms.

### 2.4. Ethylene Polymerization 

A round-bottom Schlenk flask with stirring bar was heated for 3 h at 150 °C under vacuum and then cooled to room temperature. The flask was pressurized to 1.5 atm of ethylene (absolute pressure) and vented three times. The appropriate MAO solution as a cocatalyst and toluene was added into the glass reactor under 1.5 atm of ethylene. The system was continuously stirred for 5 min, and then toluene and 1 mL of a solution of Ti complex in toluene were added sequentially by syringe to the well-stirred solution, and the total reaction volume was kept at 35 mL. The ethylene pressure was kept constant at 1.5 atm (absolute pressure) by continuous feeding of gaseous ethylene throughout the reaction. The other reaction temperatures were controlled with an external oil bath or a cooler in polymerization experiments. The polymerizations were terminated by the addition of 200 mL of acidic methanol (95:5 methanol/HCl) after continuously stirring for the desired time. The resulting precipitated polymers were collected by filtration, washed with methanol three times, and then dried in vacuum at 60 °C to a constant weight.

### 2.5. Copolymerization of Ethylene and Norbornene

A Schlenk glass flask with stirring bar was heated for 3 h at 150 °C under vacuum and then slowly cooled to room temperature. The flask was vented three times using ethylene gas. Then the glass flask was charged with toluene, the prescribed amount of MAO solution, and norbornene at initialization temperature of 30 °C. The system was continuously stirred for 5 min, and then the titanium complex solution was charged into the glass flask. The pressure of 1.5 atm (absolute pressure) was maintained by continuously feeding ethylene gas and the polymerization reaction was performed for the desired time. Polymerization was terminated by the addition of acidic methanol (methanol/HCl, 95:5). The resulting precipitated polymers were collected by filteration, washing with methanol three times, and drying under vacuum at 60 °C to a constant weight.

### 2.6. Synthesis of Bis(β-ketoamino) Titanium Complexes 

Under nitrogen, to a stirred solution of ligand (2.2 mmol) in 40 mL toluene at −78 °C was added a 2.2 M *n*-BuLi hexane solution (1.0 mL, 2.2 mmol) dropwise. The mixtures were allowed to warm to room temperature slowly and stirred for 2.5 h. TiCl_4_ (1.2 mmol) in 20.0 mL toluene was dropped into the resulting mixture at −78 °C with stirring over 0.5 h. The mixtures were allowed to warm to room temperature slowly and further stirred for 16 h. The resulting filtrate was further concentrated in vacuum to ~5 mL after filteration. Dried hexane (30 mL) was added into the concentrated solution, and the mixture was stirred for a certain time and then filtered. The residual solid was washed three times with dried hexane and dried in vacuum. Further recrystallizations of the obtained solid products in hexane/toluene solution afforded the pure titanium complexes **1**–**3**. ^1^H NMR spectroscopy of titanium complexes **1**–**3** were shown in Figures S1–S3.

Ti complex **1** ((**L1**)_2_TiCl_2_): yield: 65%. ^1^H NMR (C_6_D_6_), δ (ppm): 8.82 (d, 2H), 7.36 (t, 2H), 7.02 (t, 1H), 6.89–6.64 (m, 8H), 6.52 (d, 2H), 1.54 (s, 3H). EI–MS (*m*/*z*): 787 (M–Cl); 354 (Ligand+H). Anal. calcd. for C_46_H_36_N_6_Cl_2_TiO_2_: C, 67.08; H, 4.41; N, 10.20. Found: C, 67.58; H, 4.31; N, 10.06.

Ti complex **2** ((**L2**)_2_TiCl_2_): yield: 72%. ^1^H NMR (C_6_D_6_), δ (ppm): 8.77 (d, 2H), 7.35 (t, 2H), 7.27 (t, 2H), 7.13–6.53 (m, 6H), 6.41 (t, 2H), 2.23 (s, 3H), 1.55 (s, 3H). EI–MS (*m*/*z*): 815 (M–Cl); 368 (Ligand+H). Anal. calcd. for C_48_H_40_N_6_Cl_2_TiO_2_: C, 67.69; H, 4.73; N, 9.87. Found: C, 67.43; H, 4.61; N, 10.08.

Ti complex **3** ((**L3**)_2_TiCl_2_): yield: 80%. ^1^H NMR (C_6_D_6_), δ (ppm): 8.85 (d, 2H), 7.77(t, 1H), 7.58 (m, 1H), 7.36 (m, 3H), 7.20 (m, 2H), 7.12–6.69 (m, 9H), 6.36–6.61 (m, 2H), 1.59 (s, 3H). EI–MS (*m*/*z*): 887 (M–Cl); 404 (Ligand+H). Anal. calcd. for C_54_H_40_N_6_Cl_2_TiO_2_: C, 70.21; H, 4.36; N, 9.10. Found: C, 70.42; H, 4.24; N, 9.01.

## 3. Results and Discussion

### 3.1. Synthesis of Bis(β-ketoamino) Titanium Complexes

**Scheme 1:**
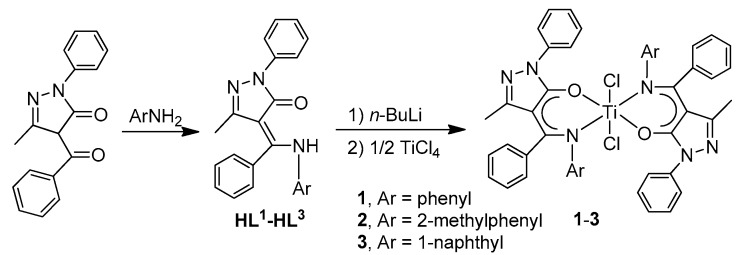
Synthesis of titanium complexes.

The synthetic route of the titanium complex is shown in Scheme 1. β-Ketoamine ligands containing pyrazolone rings were prepared following a previously reported method [57]. After the β-ketoamine ligands were treated with *n*-butyllithium in toluene, 0.5 equiv of TiCl_4_ was added to the solutions and the titanium complexes **1**–**3** were obtained as dark red solids. The structures of bis(β-ketoamino) titanium complexes **1**–**3** were confirmed by elemental analysis and EI–MS and ^1^H NMR spectroscopy. In comparison with ^1^H NMR spectra of ligand compounds **HL**, no labile protons were observed at ~13 ppm for titanium complexes (see Figures S1–S3 in Supplementary Materials), suggesting that titanium complexes were successfully synthesized.

**Figure 1:**
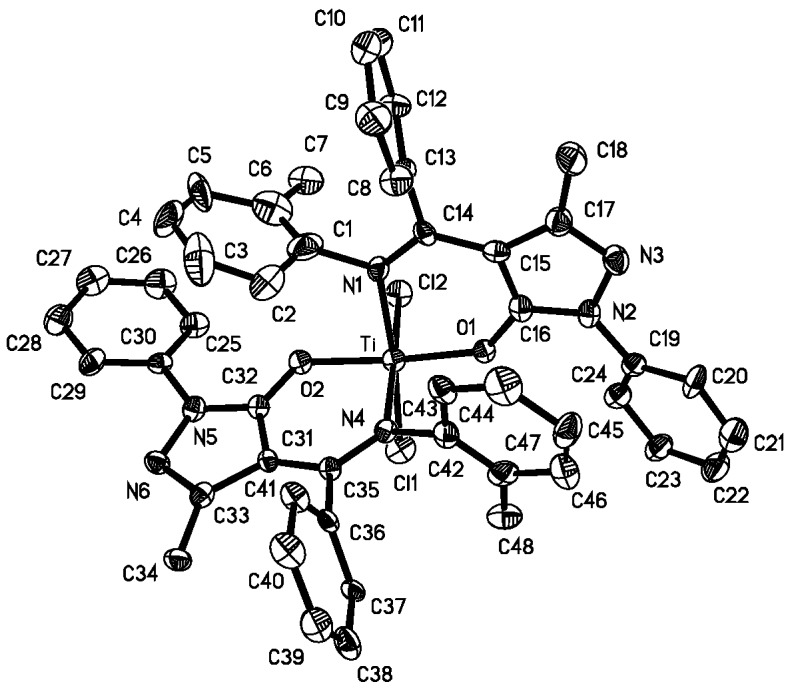
Molecular structure of Ti complex **2** with thermal ellipsoids drawn at the 30% probability level. Hydrogen atoms and toluene were omitted for clarity.

A crystal of the titanium complex 2 suitable for X-ray crystallography was grown from hexane/toluene solution by slow evaporation. The crystallographic data, data collection, and refinement are summarized in Tables S1–S5. As shown in Figure 1, the molecular structure of the titanium complex 2 belongs to the C_2_-symmetric system with a distorted octahedral geometry in the solid state. The two oxygen donor atoms are situated in the *trans* position with the O(1)–Ti–O(2) angle, of 164.93°, while the two nitrogen donor atoms are situated in the *cis* position with the N(1)–Ti–N(4) angle of 90.61°. The two chlorine atoms are situated in the *cis* position with the Cl(1)–Ti–Cl(2) angle of 92.40°. The Ti–O (1.8739 Å), Ti–N (2.209 Å), and Ti–Cl (2.2695 Å) bond distances are also typical for bis(phenoxy-imino) titanium and bis(β-enaminoketonato) titanium complexes [19,60].

### 3.2. Ethylene Homopolymerization

In the presence of MAO, bis(β-ketoamino) titanium complexes **1**–**3** were firstly investigated as precursors for ethylene polymerization. The polymerization data summarized in Table 1 clearly showed that the substituent effect of the ligand played an important role in the catalytic performances. The order of catalytic activities for ethylene polymerization is **1** > **2** > **3** at the optimized temperatures, suggesting that bulky substituent on N-aryl moiety decreased catalytic activity for ethylene polymerization. Additionally, increasing the steric hindrance of the ligand also led to a decrease in polymer molecular weight. This steric effect of *o*-aryl substituents on ethylene polymerization activity conflicts with previous observations made using bis(β-diketiminato) titanium catalysts [23]. An appropriate reason is that the crowded space around titanium metal center provided by ligands with three aryl substituents may slow down the coordination and insertion of ethylene, thus decreasing molecular weight and catalytic activity.

The polymerization results in Table 1 also demonstrated the effect of reaction temperature on ethylene polymerization. It was found that the catalytic activity increased with increasing reaction temperature and reached a maximal value. A decrease in activity was observed at higher temperature, and this was probably related to the thermal instability of the active species at higher temperatures. Molecular weight decreased uniformly with the increase of polymerization temperature, suggesting acceleration of chain transfer/termination. This is a common behavior for most of the non-metallocene Ti catalyst systems [61]. Besides, steric substituents influence the thermal stability of titanium catalysts. Although catalyst 1 was the most highly active at −10 °C, it showed the lowest activity at high temperature of 50 °C. A huge drop of 84% in activity was observed for catalyst 1 when the temperature was increased from −10 to 50 °C, whilst moderately decreased activity was observed for catalyst 3. This observation can be attributed to the protection and stabilization of the nickel metal center provided by bulky substituents. 

**Figure 2:**
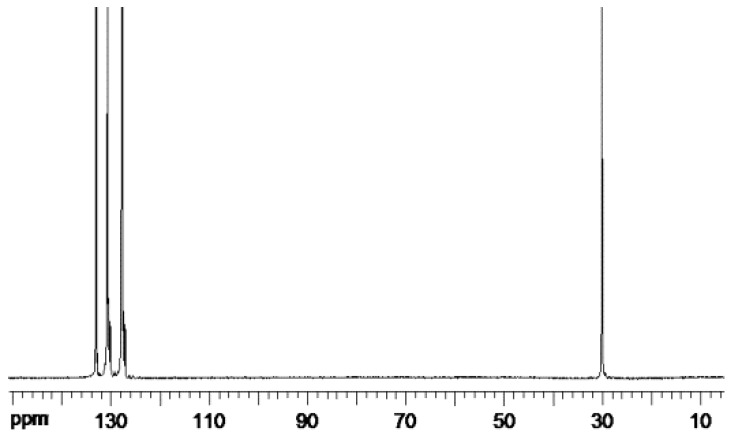
^13^C NMR spectrum of the polyethylene obtained from **1**/MAO (entry 4).

**Figure 3:**
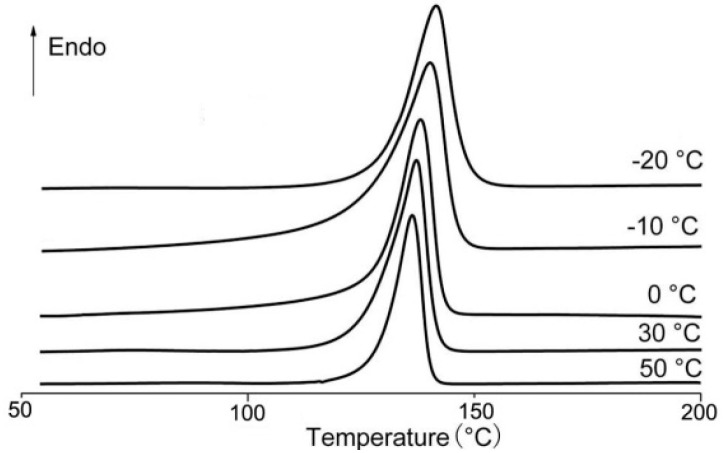
DSC curves of polyethylenes obtained by **1**/MAO at different temperatures (entries 1–5 in Table 1).

High-temperature ^13^C NMR analysis of the obtained polyethylene showed a peak at 30.0 ppm, and no signals originating from branching carbons were observed (Figure 2). The melting points (*T*_m_) of polymers measured by the differential scanning calorimetry (DSC) were in the 130–138 °C region (Figure 3), indicating that the produced polymers possessed linear structure with virtually no branching.

### 3.3. Copolymerization of Ethylene and Norbornene

Copolymerizations of ethylene and norbornene were also carried out with bis(β-ketoamino) titanium complexes **1**–**3** activated with MAO under the conditions of 30 °C and 1.5 atm ethylene pressure, and copolymerization data are summarized in Table 2. Molecular weight distributions (*M*_w_/*M*_n_) of the polymeric products are close to 2 and appear as a single modal in GPC chromatogram, which indicates that copolymerization occurs at the single active site and the polymerization products are “true” copolymers instead of the blend of homopolymers. Copolymerization results in Table 2 clearly demonstrate that the order of catalytic activities for copolymerization is **1** > **2** > **3** under the same conditions, which is same trend to ethylene homopolymerization. Less bulky catalyst **1** with phenyl groups showed the highest copolymerization activity and afforded the copolymer with the highest molecular weight. The same trend to ethylene homopolymerization can be attributed to extremely low activities of bis(β-ketoamino) titanium catalysts for norbornene polymerization. Hence, substituent effect on copolymerization activity and molecular weight is a result of steric effect. However, the highest norbornene incorporation in copolymers was achieved using catalyst **1** among three catalysts. Substituent effect on norbornene incorporation can also be interpreted to steric effect of *o*-aryl substituents, because of bulky norbornene monomer. 

Catalyst **1** was selected to further investigate the effects of the norbornene addition on copolymerization of ethylene and norbornene because of high catalytic activity. Polymerization results (entries 1–5 in Table 2) show that increasing norbornene addition from 1 to 12 g leads to a decrease in copolymerization activity and molecular weight. This resulted from extremely low activity of catalyst **1** for norbornene polymerization. However, incorporation of norbornene increased with an increase in norbornene addition and then was gradually close to a constant value of 50 mol %. Generally, the obtained E–N copolymers had similar norbornene incorporation (<50 mol %) to alternating E–N copolymers obtained by other non-metallocenes titanium catalysts [19,23].

**Figure 4:**
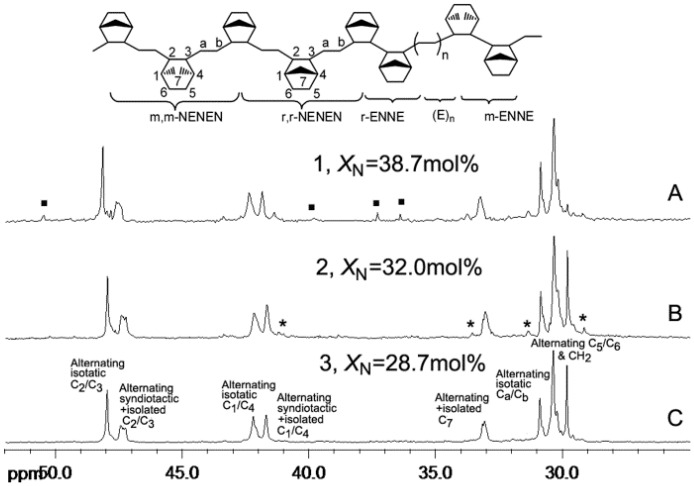
^13^C NMR spectra of the polyethylenes obtained from **1–3**/MAO at 2 g norbornene addition (entries 2, 6, and 8 in Table 2). (* represents signals of NN dyad, and ■ represents signals of NNN triad).

Microstructures of the E–N copolymers obtained at the same conditions were further determined by ^13^C NMR technique in order to investigate the effect of catalyst structure on copolymer microstructure. The ^13^C NMR spectroscopy of the copolymer containing 28.7 mol % norbornene incorporation obtained by catalyst **3** showed the “classical” resonances of alternating structure. Eight major peaks which are characteristics of alternating (ENEN) and isolated (ENEE) norbornene sequences of E–N copolymer were observed in Figure 4C. These signals were assigned according to the previous literature: 29.9, 30.4, 30.9 ppm to C_5_/C_6_ and successive ethylene sequences; 33.1 ppm to C_7_; 41.7, 42.2 ppm to C_1_/C_4_ and 47.4, 48.0 ppm to C_2_/C_3_ [19,23,25]. 

The E–N copolymers obtained by catalysts **1** and **2** displayed the higher levels of norbornene incorporation, and ^13^C NMR spectra of copolymers were slightly different. In addition to eight major peaks, several minor signals were observed. As shown in Figure 4B, several minor signals at 28.3, 31.4, 33.6, 41.2 ppm appeared for the copolymer containing 32.0 mol % norbornene incorporation obtained by catalyst **2**. As described in previous assignment of E–N copolymers, two signals at 28.3 and 31.4 ppm are assigned to carbons C_5_/C_6_ in *meso*- and *racemic*-ENNE sequences, the resonance at 33.6 ppm is assigned to C_7_ in ENN, and the resonance at 41.2 ppm is assigned to C_1_/C_4_ of *racemic*-ENNE sequence [23,25]. This strongly suggests the presence of the NN dyad sequences in the E–N copolymer is obtained by catalyst **2**. In the spectroscopy of the copolymer containing 38.7 mol % norbornene incorporation obtained by catalyst **2**, additional weak signals at 31.9, 34.2–37.6 ppm, 39.0–40.1 ppm, and 48.6–53.0 ppm can be assigned to NNN triad sequences in the E–N copolymer [23,25]. Additionally, the decreased intensity of successive ethylene sequences at 29.9 ppm was observed because more norbornene units are homogeneously distributed on the copolymer chain. 

**Figure 5:**
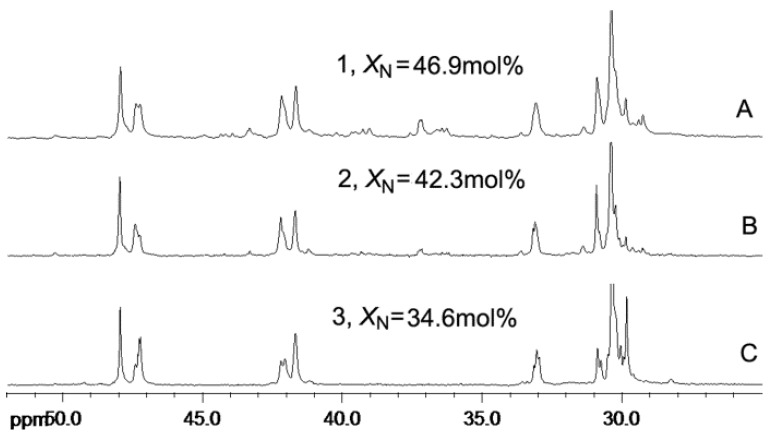
^13^C NMR spectra of the polyethylenes obtained from **1**–**3**/MAO at 4 g norbornene addition (entries 3, 7, and 9 in Table 2).

Increased norbornene addition from 2 to 4 g can improve norbornene incorporation in copolymer. Though norbornene incorporation increased, bulky catalyst **3** still afforded the alternating E–N copolymers without NN dyad sequences (Figure 5C). For the E–N copolymers obtained by catalyst **1** and **2**, more intensive NN dyad and NNN triad resonances appeared (Figure 5A,B). Hence, *o*-substituent of ligand predominantly determines copolymer microstructure.

**Figure 6:**
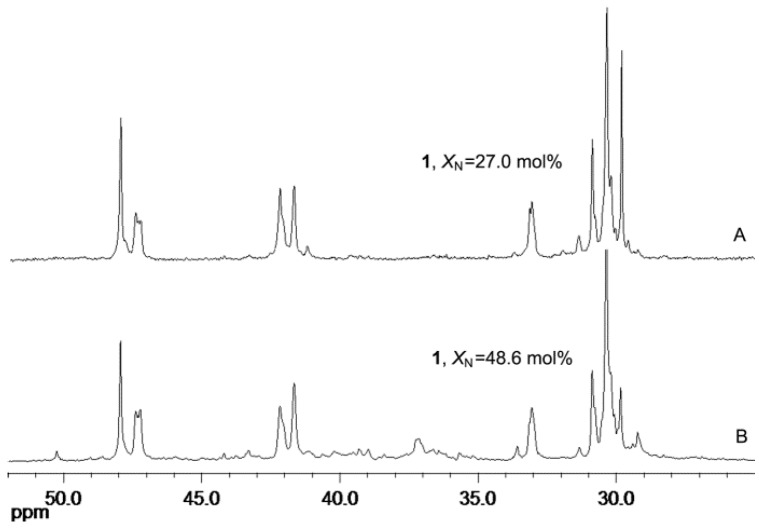
^13^C NMR spectra of the polyethylenes obtained from **1**/MAO at different norbornene additions (entries 1 and 5 in Table 2).

Catalyst **1** was chosen to investigate the effect of norbornene addition on copolymer microstructure. As shown in Figure 6A, the E–N copolymer that obtained low norbornene addition of 1 g had 27.0 mol % norbornene incorporation, and only displayed eight characteristic signals of alternating (ENEN) and isolated (ENEE) norbornene sequences. With an increase in norbornene addition, norbornene incorporation increased and NN dyad sequences appeared in the copolymer (Figure 5A and Figure 6A). For the copolymer that obtained 12 g of norbornene addition with the highest norbornene incorporation of 48.6%, more intensive resonances assigned to the NN dyad and NNN triad sequences appeared in the E–N copolymer (Figure 6B) [23,25]. This suggests that increased norbornene addition not only improved norbornene incorporation in copolymer, but also enhanced insertion frequency of norbornene into the Ti–N bond, thus forming successive norbornene sequences.

The E–N copolymers obtained by bis(β-ketoamino) titanium catalysts had a glass transition temperature (*T*_g_) in a range from 58–115 °C, determined by means of the differential scanning calorimetry (DSC) analysis. The *T*_g_ value of 58 °C was detected for the copolymer with 27.0 mol % norbornene incorporation, while the highest *T*_g_ value of 115 °C was determined for the copolymer with 48.6 mol % norbornene incorporation. The *T*_g_ value was closely dependent upon norbornene incorporation in the copolymers, and the *T*_g_ value increased with increasing norbornene incorporation in copolymers. 

## 4. Conclusions

In this study, we have successfully synthesized and characterized novel bis(β-ketoamino) titanium complexes containing pyrazolone rings **1**–**3**. Ethylene polymerization using **1**–**3** activated with MAO produced linear polyethylene with high activities. The substituent influence on ethylene polymerization was largely attributed to the steric effect of *o*-aryl substituents. Copolymerizations of ethylene and norbornen using catalyst **1**–**3**/MAO produced E–N copolymers with high acitvities. The substituent influence on copolymerization activity, copolymer molecular weight, and norbornene incorporation was attributted to steric effect. Although increased norbornene addition improved norbornene incorporation in the copolymers, *o*-substituents of ligand predominantly determined copolymer microstructure. All of the copolymers showed the *T*_g_ in a temperature range from 58 to 115 °C, which increased with additional incorporation of norbornene. The bis(β-ketoamino) titanium complex containing pyrazolone rings is a promising candidate for tuning the microstructure of E–N copolymers with the design of the *o*-substituents on the aryl ring.

## Figures and Tables

**Table 1:** Ethylene polymerizations with **1**–**3**/ methylaluminoxane (MAO).

Entry	Cat.	*T*_p_ (°C)	Yield (g)	Activity (kg PE /(mol·Ti·h))	*M*_w_^a^ (kg/mol)	*M*_w_/*M*_n_^a^	*T*_m_^b^ (°C)
1	**1**	−20	0.61	244	715	1.45	138
2	**1**	−10	1.48	592	650	1.56	137
3	**1**	0	1.11	444	556	1.61	136
4	**1**	30	0.65	260	240	1.71	135
5	**1**	50	0.26	104	160	1.82	130
6	**2**	−20	0.52	208	614	1.41	138
7	**2**	−10	1.42	568	590	1.49	137
8	**2**	0	1.17	468	435	1.52	136
9	**2**	30	0.98	392	345	1.65	135
10	**2**	50	0.45	180	285	1.76	132
11	**3**	−20	0.25	100	588	1.49	138
12	**3**	−10	0.39	156	504	1.50	135
13	**3**	0	0.73	292	405	1.63	134
14	**3**	30	0.56	224	340	1.77	132
15	**3**	50	0.41	164	290	1.84	131

Polymerization conditions: Ti complex, 5 μmol; Al/Ti = 200; ethylene pressure, 1.5 atm; reaction time, 30 min, solvent, toluene; total volume: 35 mL; ^a^ Determined by high temperature gel permeation chromatography (GPC) in 1,2,4-trichlorobenzene at 150 °C; ^b^ Determined by differential scanning calorimetry (DSC).

**Table 2:** Copolymerizations of ethylene and norbornene with **1–3**/MAO.

Entry	Cat.	NB Addition (g)	Yield (g)	Activity ^a^	*M*_w_^b^ (kg/mol)	*M*_w_/*M*_n_^b^	*X*_N_^c^ mol %	*T*_g_ (°C)
1	**1**	1	1.47	147	531	1.68	27.0	58
2	**1**	2	1.10	110	449	1.74	38.7	89
3	**1**	4	0.74	74	372	1.86	46.9	97
4	**1**	8	0.39	39	270	1.78	47.4	106
5	**1**	12	0.24	24	184	1.80	48.6	115
6	**2**	2	0.90	90	379	1.90	32.0	60
7	**2**	4	0.64	64	303	2.02	42.3	91
8	**3**	2	0.55	55	312	1.95	28.7	46
9	**3**	4	0.48	48	264	2.14	34.6	71

Polymerization conditions: Ti complex, 10 μmol; Al/Ti = 200; reaction time, 60 min, temperature, 30 °C, solvent, toluene; total volume: 35 mL. ^a^ In unit of (kg P(E–N) (mol·Ti·h)^−1^); ^b^ Determined by high temperature gel permeation chromatography (GPC) in 1,2,4-trichlorobenzene at 150 °C; ^c^ Determined by ^13^C NMR.

## Supplementary Materials

Supplementary Materials are available online at www.mdpi.com/2073-4360/9/7/262/s1.
