# Supplementary material for: Homo- and Copolymerizations of Ethylene and Norbornene Using Bis(β-ketoamino) Titanium Catalysts Containing Pyrazolone Rings
Source: Polymers (Basel). 2017 Jun 30;9(7):262. doi: 10.3390/polym9070262 (PMC6432339; doi:10.3390/polym9070262)
Supplement: Supplementary file 1 [file polymers-09-00262-s001.pdf]

Supporting Information

Homo- and Copolymerizations of Ethylene and Norbornene Using Bis(*β*-ketoamino) Titanium Catalysts Containing Pyrazolone Rings

Lixia Pei[1,2]*, San He, Jie Gao*[3]*, Heng Liao*[3]*, and Haiyang Gao**[3]

1 State Key Laboratory of Oil and Gas Reservoir Geology and Exploitation, Southwest Petroleum University, Chengdu 610550, China

2 School of Chemistry and Chemical Engineering, South China University of Technology, Guangzhou 510641, China

3 School of Materials Science and Engineering, PCFM Lab, GD HPPC Lab, Sun Yat-sen University, Guangzhou 510275, China.

*Corresponding author. Fax: +86-20-84114033. Tel.: +86-20-84113250. Email: Gao Haiyang; gaohy@mail.sysu.edu.cn

(1) ^{1}H NMR spectra of ligand and Ti complexes.

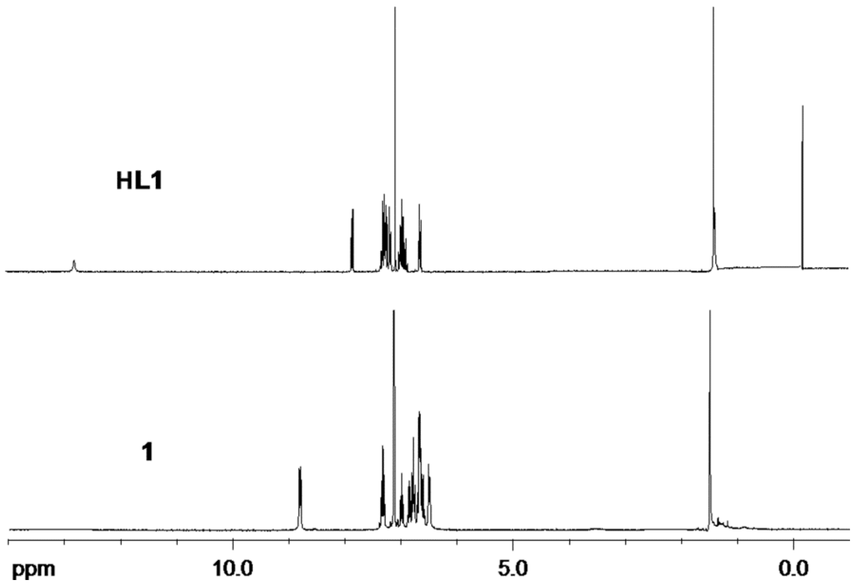

Figure S1. ^{1}H NMR spectra of ligand **HL1** and complex **1** at room temperature.

Ligand **HL1** ^{1}H NMR ($CDCl_3$), δ(ppm): 12.99 (s, 1H, -NH); 8.04 (d, 2H), 7.49-7.32 (m, 7H), 7.14 (t, 3H), 7.06 (t, 1H), 6.81 (d, 2H), 1.56 (s, 3H).

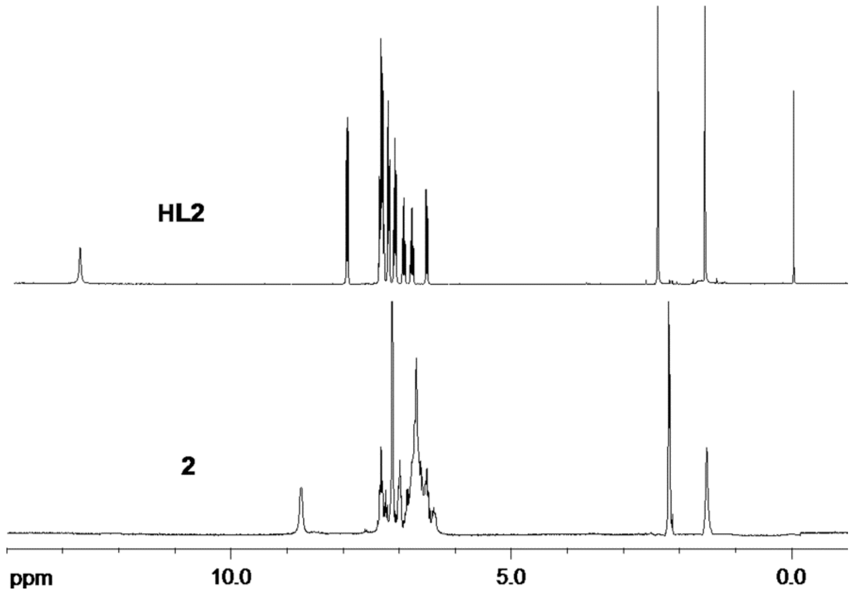

Figure S2. ^{1}H NMR spectra of ligand HL2 and complex 2 at room temperature.

Ligand **HL2** ^{1}H NMR ($CDCl_3$), δ (ppm): 12.79 (s, 1H, -NH); 8.01 (d, 2H), 7.42-7.24 (m, 7H), 7.15 (t, 2H), 6.99 (t, 1H), 6.84 (t, 1H), 6.59(d, 1H), 2.44(s, 3H), 1.59 (s, 3H).

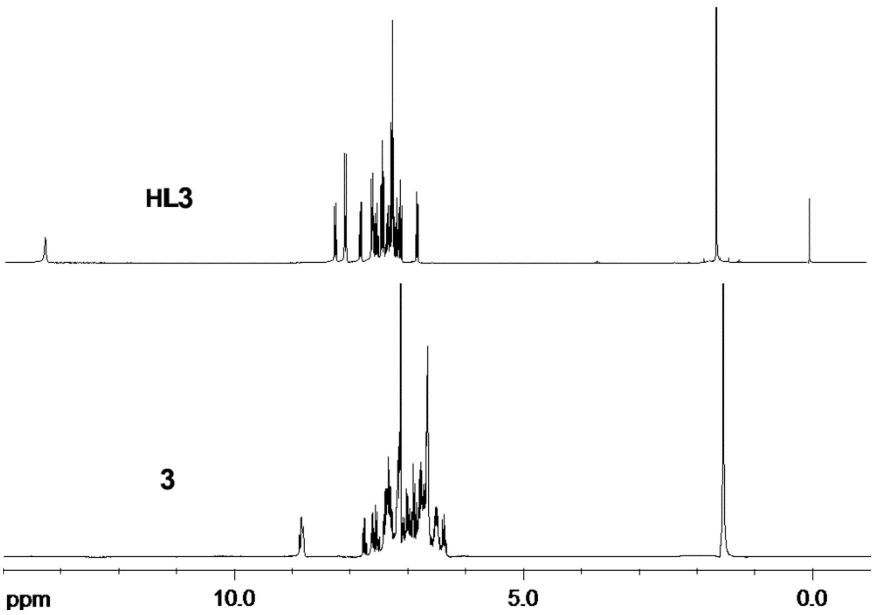

Figure S3. ^{1}H NMR spectra of ligand **HL3** and complex **3** at room temperature.

Ligand **HL3** ^{1}H NMR ($CDCl_3$), δ (ppm): 12.91 (s, 1H, -NH); 8.02 (d, 2H), 7.48-7.30 (m, 7H), 7.15 (t, 1H), 6.93 (d, 2H), 6.68 (d, 2H), 2.23(s, 3H), 1.57 (s, 3H).

(2) Crystal data and structure refinement for 2

Table S1. Crystallographic data and structure refinement for Ti complex **2**.

	**2**
Empirical formula	$C_{55}H_{48}Cl_2N_6O_2Ti$
Fw	943.8
T (K)	293(2)
Crystal system	Monoclinic
Space group	c2/c
a (Å)	18.3877(7)
b (Å)	15.8609(7)
c (Å)	16.8067(6)
α (°)	90
β (°)	100.288(4)
γ (°)	90
V (Å^3)	4822.8(3)
Z	4
D_{calc}(Mg/m^3)	1.304
Absorption coefficient (mm^{-1})	0.337
F(000)	1980
θ range (°)	2.78 to 27.00
Reflections collected	29635
Unique reflections	10426
Completeness to θ (%)	99.0 (θ = 27.00)
Data / restraints / parameters	10426 / 2 / 595
Goodness-of-fit on F^2	0.986
Final R indices [I >2σ (I)]	R_1 = 0.0837, wR_2 = 0.2304
Largest difference in peak and hole (e Å^{-3})	1.689 and -0.592

Table S2. Atomic coordinates (x 10^4) and equivalent isotropic displacement parameters (A^2 x 10^3) for **2**. U(eq) is defined as one third of the trace of the orthogonalized Uij tensor.

	x	y	z	U(eq)
Ti	12360(1)	8042(1)	2706(1)	34(1)
Cl(1)	11821(1)	9131(1)	1934(1)	55(1)
Cl(2)	12874(1)	7511(1)	1669(1)	46(1)
C(1)	13677(3)	7265(4)	3980(5)	79(2)
C(2)	13684(3)	7714(4)	4835(3)	58(1)
C(3)	14320(4)	7909(5)	5245(4)	97(2)
C(4)	14983(3)	7816(5)	4925(6)	105(3)
C(5)	14995(4)	7467(5)	4206(5)	90(3)
C(6)	14285(5)	7154(4)	3740(4)	91(2)
C(7)	14319(4)	6773(4)	2985(3)	74(2)
C(8)	13141(2)	5754(3)	4348(3)	39(1)
C(9)	13653(3)	5207(3)	4142(3)	54(1)
C(10)	14060(3)	4692(4)	4726(4)	67(2)
C(11)	13951(3)	4716(4)	5499(4)	70(2)
C(12)	13435(3)	5269(4)	5714(3)	67(2)
C(13)	13027(3)	5772(3)	5148(3)	54(1)
C(14)	12691(2)	6325(3)	3738(2)	37(1)
C(15)	11959(2)	6060(3)	3408(3)	37(1)
C(16)	11456(2)	6536(3)	2867(2)	33(1)
C(17)	11534(3)	5314(3)	3532(3)	46(1)
C(18)	11735(3)	4573(4)	4067(4)	77(2)
C(19)	10119(2)	6381(3)	2263(2)	37(1)
C(20)	9474(2)	6108(4)	2505(3)	51(1)
C(21)	8808(3)	6423(4)	2125(4)	67(2)
C(22)	8766(3)	6987(4)	1506(4)	63(2)
C(23)	9395(3)	7230(4)	1234(3)	58(1)
C(24)	10084(3)	6926(3)	1616(3)	47(1)
C(25)	14607(3)	9261(3)	2747(3)	49(1)
C(26)	15307(3)	9161(4)	2529(4)	64(2)
C(27)	15912(3)	9552(4)	2952(3)	59(2)
C(28)	15847(3)	10044(4)	3595(4)	65(2)
C(29)	15166(2)	10151(3)	3826(3)	51(1)
C(30)	14560(2)	9761(3)	3407(3)	37(1)
C(31)	12770(2)	9699(3)	4022(2)	31(1)
C(32)	13263(2)	9370(3)	3556(2)	33(1)
C(33)	13128(2)	10436(3)	4388(3)	36(1)
C(34)	12885(2)	11082(3)	4931(3)	46(1)
C(35)	12124(2)	9264(3)	4145(2)	31(1)

C(36)	11750(2)	9571(3)	4813(2)	33(1)
C(37)	11144(2)	10100(3)	4676(3)	40(1)
C(38)	10861(3)	10406(3)	5325(3)	51(1)
C(39)	11159(3)	10182(3)	6097(3)	54(1)
C(40)	11765(3)	9652(3)	6243(3)	53(1)
C(41)	12062(2)	9358(3)	5595(3)	40(1)
C(42)	11296(2)	8102(3)	3995(3)	46(1)
C(43)	11503(3)	7540(3)	4674(3)	46(1)
C(44)	10963(3)	7041(4)	4904(3)	64(2)
C(45)	10241(3)	7098(4)	4492(4)	65(2)
C(46)	10051(3)	7644(4)	3889(4)	59(2)
C(47)	10578(3)	8168(3)	3604(3)	48(1)
C(48)	10349(3)	8748(3)	2928(3)	54(1)
C(49)	12225(6)	13199(7)	5795(5)	141(4)
C(50)	12559(4)	12805(5)	6599(5)	85(2)
C(51)	13088(4)	13183(5)	7184(5)	90(2)
C(52)	13356(5)	12776(6)	7910(5)	103(3)
C(53)	13063(5)	12032(6)	8092(5)	93(2)
C(54)	12500(5)	11641(7)	7516(6)	112(3)
C(55)	12282(4)	12050(5)	6796(5)	88(2)
N(1)	12954(2)	7040(2)	3531(2)	36(1)
N(2)	10817(2)	6114(2)	2704(2)	38(1)
N(3)	10869(2)	5362(3)	3109(2)	50(1)
N(4)	11888(2)	8583(2)	3726(2)	32(1)
N(5)	13866(2)	9873(2)	3665(2)	36(1)
N(6)	13779(2)	10538(2)	4184(2)	41(1)
O(1)	11562(1)	7279(2)	2572(2)	35(1)
O(2)	13202(1)	8697(2)	3122(2)	36(1)

Table S3. Bond lengths [A] and angles [deg] for **2**.

Ti-O(1)	1.885(3)
Ti-O(2)	1.891(3)
Ti-N(4)	2.227(3)
Ti-N(1)	2.259(4)
Ti-Cl(1)	2.2777(15)
Ti-Cl(2)	2.2875(12)
C(1)-C(6)	1.267(9)
C(1)-N(1)	1.451(7)
C(1)-C(2)	1.602(10)
C(2)-C(3)	1.285(8)
C(3)-C(4)	1.425(7)

C(4)-C(5)	1.334(8)
C(5)-C(6)	1.482(10)
C(6)-C(7)	1.417(8)
C(8)-C(9)	1.370(6)
C(8)-C(13)	1.397(6)
C(8)-C(14)	1.501(6)
C(9)-C(10)	1.389(8)
C(10)-C(11)	1.349(8)
C(11)-C(12)	1.386(8)
C(12)-C(13)	1.360(7)
C(14)-N(1)	1.304(6)
C(14)-C(15)	1.425(6)
C(15)-C(16)	1.396(6)
C(15)-C(17)	1.453(6)
C(16)-O(1)	1.307(5)
C(16)-N(2)	1.338(5)
C(17)-N(3)	1.303(6)
C(17)-C(18)	1.486(7)
C(19)-C(24)	1.383(7)
C(19)-C(20)	1.390(6)
C(19)-N(2)	1.426(5)
C(20)-C(21)	1.371(7)
C(21)-C(22)	1.364(8)
C(22)-C(23)	1.372(8)
C(23)-C(24)	1.400(7)
C(25)-C(30)	1.379(6)
C(25)-C(26)	1.410(6)
C(26)-C(27)	1.357(8)
C(27)-C(28)	1.356(8)
C(28)-C(29)	1.385(7)
C(29)-C(30)	1.357(6)
C(30)-N(5)	1.430(5)
C(31)-C(32)	1.400(5)
C(31)-C(35)	1.421(5)
C(31)-C(33)	1.426(6)
C(32)-O(2)	1.286(5)
C(32)-N(5)	1.353(5)
C(33)-N(6)	1.312(5)
C(33)-C(34)	1.492(6)
C(35)-N(4)	1.320(5)
C(35)-C(36)	1.498(5)
C(36)-C(41)	1.379(6)
C(36)-C(37)	1.381(6)
C(37)-C(38)	1.378(6)

C(38)-C(39)	1.361(7)
C(39)-C(40)	1.383(7)
C(40)-C(41)	1.383(6)
C(42)-C(47)	1.369(7)
C(42)-C(43)	1.445(7)
C(42)-N(4)	1.465(5)
C(43)-C(44)	1.379(7)
C(44)-C(45)	1.386(8)
C(45)-C(46)	1.331(8)
C(46)-C(47)	1.424(7)
C(47)-C(48)	1.463(7)
C(49)-C(50)	1.516(10)
C(50)-C(55)	1.367(10)
C(50)-C(51)	1.388(10)
C(51)-C(52)	1.391(11)
C(52)-C(53)	1.354(11)
C(53)-C(54)	1.427(12)
C(54)-C(55)	1.368(11)
N(2)-N(3)	1.370(5)
N(5)-N(6)	1.395(5)
O(1)-Ti-O(2)	164.93(13)
O(1)-Ti-N(4)	86.40(12)
O(2)-Ti-N(4)	84.87(12)
O(1)-Ti-N(1)	84.38(13)
O(2)-Ti-N(1)	83.45(13)
N(4)-Ti-N(1)	90.61(13)
O(1)-Ti-Cl(1)	99.66(10)
O(2)-Ti-Cl(1)	92.22(10)
N(4)-Ti-Cl(1)	87.53(10)
N(1)-Ti-Cl(1)	175.43(10)
O(1)-Ti-Cl(2)	95.19(9)
O(2)-Ti-Cl(2)	93.53(9)
N(4)-Ti-Cl(2)	178.39(10)
N(1)-Ti-Cl(2)	89.33(10)
Cl(1)-Ti-Cl(2)	92.40(5)
C(6)-C(1)-N(1)	125.4(8)
C(6)-C(1)-C(2)	118.8(6)
N(1)-C(1)-C(2)	115.8(5)
C(3)-C(2)-C(1)	116.6(5)
C(2)-C(3)-C(4)	122.1(7)
C(5)-C(4)-C(3)	122.6(8)
C(4)-C(5)-C(6)	117.5(8)
C(1)-C(6)-C(7)	121.8(8)

C(1)-C(6)-C(5)	122.0(7)
C(7)-C(6)-C(5)	116.1(7)
C(9)-C(8)-C(13)	118.9(5)
C(9)-C(8)-C(14)	122.0(4)
C(13)-C(8)-C(14)	119.1(4)
C(8)-C(9)-C(10)	120.2(5)
C(11)-C(10)-C(9)	120.6(5)
C(10)-C(11)-C(12)	119.7(5)
C(13)-C(12)-C(11)	120.4(5)
C(12)-C(13)-C(8)	120.2(5)
N(1)-C(14)-C(15)	121.5(4)
N(1)-C(14)-C(8)	121.4(4)
C(15)-C(14)-C(8)	117.1(4)
C(16)-C(15)-C(14)	124.3(4)
C(16)-C(15)-C(17)	103.0(4)
C(14)-C(15)-C(17)	132.7(4)
O(1)-C(16)-N(2)	123.9(4)
O(1)-C(16)-C(15)	127.5(4)
N(2)-C(16)-C(15)	108.6(4)
N(3)-C(17)-C(15)	110.5(4)
N(3)-C(17)-C(18)	118.9(4)
C(15)-C(17)-C(18)	130.5(4)
C(24)-C(19)-C(20)	120.2(4)
C(24)-C(19)-N(2)	120.4(4)
C(20)-C(19)-N(2)	119.5(4)
C(21)-C(20)-C(19)	119.3(5)
C(22)-C(21)-C(20)	121.3(5)
C(21)-C(22)-C(23)	120.1(5)
C(22)-C(23)-C(24)	120.0(5)
C(19)-C(24)-C(23)	119.1(5)
C(30)-C(25)-C(26)	117.7(5)
C(27)-C(26)-C(25)	120.9(5)
C(28)-C(27)-C(26)	120.1(5)
C(27)-C(28)-C(29)	120.4(5)
C(30)-C(29)-C(28)	120.0(5)
C(29)-C(30)-C(25)	121.0(4)
C(29)-C(30)-N(5)	118.7(4)
C(25)-C(30)-N(5)	120.2(4)
C(32)-C(31)-C(35)	122.8(4)
C(32)-C(31)-C(33)	104.6(3)
C(35)-C(31)-C(33)	132.0(4)
O(2)-C(32)-N(5)	123.5(4)
O(2)-C(32)-C(31)	128.9(4)
N(5)-C(32)-C(31)	107.5(4)

N(6)-C(33)-C(31)	111.1(4)
N(6)-C(33)-C(34)	117.5(4)
C(31)-C(33)-C(34)	131.4(4)
N(4)-C(35)-C(31)	121.6(3)
N(4)-C(35)-C(36)	121.0(3)
C(31)-C(35)-C(36)	117.3(4)
C(41)-C(36)-C(37)	119.6(4)
C(41)-C(36)-C(35)	117.7(4)
C(37)-C(36)-C(35)	122.5(4)
C(38)-C(37)-C(36)	119.3(4)
C(39)-C(38)-C(37)	121.2(5)
C(38)-C(39)-C(40)	120.2(4)
C(39)-C(40)-C(41)	118.9(5)
C(36)-C(41)-C(40)	120.8(5)
C(47)-C(42)-C(43)	121.5(4)
C(47)-C(42)-N(4)	121.2(5)
C(43)-C(42)-N(4)	117.3(4)
C(44)-C(43)-C(42)	118.3(5)
C(43)-C(44)-C(45)	119.8(6)
C(46)-C(45)-C(44)	121.3(6)
C(45)-C(46)-C(47)	122.3(5)
C(42)-C(47)-C(46)	116.7(5)
C(42)-C(47)-C(48)	122.7(5)
C(46)-C(47)-C(48)	120.6(5)
C(55)-C(50)-C(51)	116.8(8)
C(55)-C(50)-C(49)	117.9(8)
C(51)-C(50)-C(49)	125.0(8)
C(50)-C(51)-C(52)	120.9(8)
C(53)-C(52)-C(51)	120.7(8)
C(52)-C(53)-C(54)	119.7(9)
C(55)-C(54)-C(53)	117.2(9)
C(50)-C(55)-C(54)	124.5(9)
C(14)-N(1)-C(1)	115.1(4)
C(14)-N(1)-Ti	127.9(3)
C(1)-N(1)-Ti	116.3(3)
C(16)-N(2)-N(3)	110.7(4)
C(16)-N(2)-C(19)	129.4(4)
N(3)-N(2)-C(19)	119.5(4)
C(17)-N(3)-N(2)	107.3(4)
C(35)-N(4)-C(42)	116.9(3)
C(35)-N(4)-Ti	126.1(3)
C(42)-N(4)-Ti	116.8(3)
C(32)-N(5)-N(6)	110.3(3)
C(32)-N(5)-C(30)	129.8(4)

N(6)-N(5)-C(30)	119.5(3)
C(33)-N(6)-N(5)	106.5(3)
C(16)-O(1)-Ti	134.4(3)
C(32)-O(2)-Ti	130.3(3)

Table S4. Anisotropic displacement parameters (A^2 x 10^3) for **2**. The anisotropic displacement factor exponent takes the form: -2 pi^2 [h^2 a*^2 U11 + ... + 2 h k a* b* U12]

	U11	U22	U33	U23	U13	U12
Ti	38(1)	38(1)	29(1)	-3(1)	13(1)	-2(1)
Cl(1)	59(1)	65(1)	45(1)	8(1)	15(1)	5(1)
Cl(2)	50(1)	62(1)	32(1)	-8(1)	18(1)	-3(1)
C(1)	54(3)	60(4)	120(6)	43(4)	8(4)	11(3)
C(2)	52(3)	72(4)	51(3)	18(3)	16(3)	5(3)
C(3)	103(6)	110(6)	73(5)	-3(4)	3(4)	-25(5)
C(4)	40(3)	98(6)	168(9)	34(6)	-3(5)	-12(3)
C(5)	77(5)	78(5)	91(5)	30(4)	-51(4)	-35(4)
C(6)	143(7)	68(4)	72(5)	6(4)	48(5)	8(4)
C(7)	99(5)	64(4)	56(4)	6(3)	10(3)	13(3)
C(8)	39(2)	35(3)	42(3)	-5(2)	-1(2)	1(2)
C(9)	55(3)	50(3)	59(3)	5(3)	16(3)	7(3)
C(10)	49(3)	58(4)	95(5)	15(4)	15(3)	10(3)
C(11)	66(4)	61(4)	73(4)	18(3)	-13(3)	1(3)
C(12)	89(4)	61(4)	44(3)	2(3)	-5(3)	6(3)
C(13)	66(3)	57(3)	36(3)	-3(3)	5(2)	10(3)
C(14)	43(2)	39(3)	29(2)	-11(2)	9(2)	-1(2)
C(15)	41(2)	39(3)	31(2)	0(2)	9(2)	4(2)
C(16)	36(2)	37(3)	28(2)	-9(2)	12(2)	-3(2)
C(17)	43(3)	48(3)	47(3)	-1(2)	8(2)	-6(2)
C(18)	71(4)	55(4)	97(5)	23(4)	-4(3)	-19(3)
C(19)	35(2)	48(3)	30(2)	-11(2)	7(2)	3(2)
C(20)	40(3)	73(4)	41(3)	-13(3)	11(2)	-11(2)
C(21)	40(3)	100(5)	65(4)	-34(4)	20(3)	-14(3)
C(22)	37(3)	75(4)	74(4)	-24(4)	-1(3)	8(3)
C(23)	49(3)	61(4)	57(3)	1(3)	-7(3)	1(3)
C(24)	43(3)	55(3)	45(3)	-10(3)	12(2)	-5(2)
C(25)	47(3)	54(3)	49(3)	-3(3)	21(2)	-6(2)
C(26)	64(3)	71(4)	69(4)	-4(3)	42(3)	-2(3)
C(27)	44(3)	73(4)	69(4)	12(3)	31(3)	6(3)
C(28)	38(3)	88(5)	70(4)	-2(3)	14(3)	-6(3)

C(29)	44(3)	66(4)	44(3)	-6(3)	14(2)	-6(2)
C(30)	36(2)	44(3)	33(2)	8(2)	13(2)	-2(2)
C(31)	31(2)	30(2)	30(2)	-3(2)	5(2)	-1(2)
C(32)	37(2)	32(2)	30(2)	-2(2)	7(2)	-1(2)
C(33)	32(2)	40(3)	37(2)	5(2)	7(2)	-2(2)
C(34)	40(2)	40(3)	59(3)	-9(2)	13(2)	-1(2)
C(35)	33(2)	33(2)	27(2)	-1(2)	7(2)	2(2)
C(36)	37(2)	31(2)	31(2)	-8(2)	9(2)	-5(2)
C(37)	38(2)	41(3)	41(3)	-11(2)	9(2)	-3(2)
C(38)	44(3)	52(3)	62(3)	-18(3)	21(2)	-1(2)
C(39)	63(3)	60(3)	46(3)	-14(3)	32(3)	-11(3)
C(40)	66(3)	61(3)	35(3)	-3(2)	22(2)	-15(3)
C(41)	47(3)	40(3)	37(3)	2(2)	15(2)	-3(2)
C(42)	43(3)	50(3)	48(3)	-17(2)	20(2)	-4(2)
C(43)	44(2)	50(3)	48(3)	-11(2)	21(2)	-6(2)
C(44)	81(4)	70(4)	50(3)	-3(3)	34(3)	-2(3)
C(45)	55(3)	84(5)	62(4)	-24(4)	30(3)	-12(3)
C(46)	57(3)	67(4)	60(4)	-29(3)	34(3)	-14(3)
C(47)	48(3)	52(3)	45(3)	-14(3)	16(2)	6(2)
C(48)	49(3)	61(4)	52(3)	-15(3)	12(2)	9(2)
C(49)	189(10)	124(8)	121(8)	55(6)	53(7)	41(7)
C(50)	87(5)	76(5)	96(5)	6(4)	33(4)	6(4)
C(51)	85(5)	95(6)	93(5)	-24(5)	28(4)	-8(4)
C(52)	110(6)	104(7)	93(6)	-39(6)	9(5)	15(5)
C(53)	107(6)	85(6)	92(6)	-11(5)	34(5)	20(5)
C(54)	135(7)	119(7)	93(6)	4(6)	49(6)	24(6)
C(55)	84(5)	77(5)	112(6)	-15(5)	43(4)	8(4)
N(1)	32(2)	38(2)	35(2)	-5(2)	3(2)	-4(2)
N(2)	39(2)	45(2)	31(2)	-3(2)	12(2)	-5(2)
N(3)	51(2)	41(2)	57(3)	-1(2)	6(2)	-16(2)
N(4)	34(2)	35(2)	29(2)	-1(2)	9(2)	-3(2)
N(5)	36(2)	39(2)	36(2)	0(2)	11(2)	1(2)
N(6)	40(2)	35(2)	49(2)	-7(2)	14(2)	-7(2)
O(1)	35(2)	42(2)	30(2)	-6(1)	12(1)	1(1)
O(2)	35(2)	42(2)	35(2)	-6(2)	13(1)	-3(1)

Table S5. Hydrogen coordinates (x 10^4) and isotropic displacement parameters (A^2 x 10^3) for **2**.

	x	y	z	U(eq)
H(2A)	13391	8225	4742	69
H(2B)	13440	7343	5164	69

H(3A)	14349	8118	5767	116
H(4A)	15424	8007	5230	126
H(5A)	15432	7421	4003	108
H(7A)	13834	6592	2735	110
H(7B)	14644	6295	3068	110
H(7C)	14503	7174	2642	110
H(9A)	13728	5179	3610	65
H(10A)	14412	4327	4584	80
H(11A)	14220	4363	5885	84
H(12A)	13366	5295	6248	80
H(13A)	12672	6130	5294	64
H(18A)	11324	4191	4011	115
H(18B)	11859	4757	4619	115
H(18C)	12153	4291	3917	115
H(20A)	9494	5716	2920	61
H(21A)	8377	6250	2293	81
H(22A)	8310	7207	1267	76
H(23A)	9363	7597	798	69
H(24A)	10513	7089	1435	57
H(25A)	14191	8998	2456	58
H(26A)	15356	8822	2089	77
H(27A)	16370	9483	2800	71
H(28A)	16262	10310	3884	77
H(29A)	15125	10489	4267	61
H(34A)	13266	11498	5068	69
H(34B)	12792	10815	5416	69
H(34C)	12441	11349	4659	69
H(37A)	10929	10249	4151	48
H(38A)	10459	10771	5235	62
H(39A)	10955	10387	6526	64
H(40A)	11970	9495	6768	63
H(41A)	12477	9012	5688	48
H(43A)	11989	7515	4949	55
H(44A)	11082	6667	5333	77
H(45A)	9881	6748	4640	78
H(46A)	9557	7685	3645	70
H(48A)	10772	9051	2818	81
H(48B)	9993	9139	3066	81
H(48C)	10132	8434	2457	81
H(49A)	11868	12821	5500	212
H(49B)	12608	13307	5488	212
H(49C)	11988	13720	5888	212
H(51A)	13265	13716	7089	108
H(52A)	13740	13017	8274	124

H(53A)	13227	11778	8591	111
H(54A)	12291	11129	7625	135
H(55A)	11919	11796	6415	106
H(3B)	10526	4989	3089	60
H(6A)	14090	10936	4338	49
